# Bumps in Small-World Networks

**DOI:** 10.3389/fncom.2016.00053

**Published:** 2016-06-15

**Authors:** Carlo R. Laing

**Affiliations:** Institute of Natural and Mathematical Sciences, Massey UniversityAuckland, New Zealand

**Keywords:** Ott/Antonsen, theta neuron, bump, small-world, working memory, bifurcation

## Abstract

We consider a network of coupled excitatory and inhibitory theta neurons which is capable of supporting stable spatially-localized “bump” solutions. We randomly add long-range and simultaneously remove short-range connections within the network to form a small-world network and investigate the effects of this rewiring on the existence and stability of the bump solution. We consider two limits in which continuum equations can be derived; bump solutions are *fixed points* of these equations. We can thus use standard numerical bifurcation analysis to determine the stability of these bumps and to follow them as parameters (such as rewiring probabilities) are varied. We find that under some rewiring schemes bumps are quite robust, whereas in other schemes they can become unstable via Hopf bifurcation or even be destroyed in saddle-node bifurcations.

## 1. Introduction

Spatially-localized “bumps” of activity in neuronal networks have been studied for many years, as they are thought to play a role in short term memory (Camperi and Wang, 1998; Compte et al., 2000; Bressloff, 2012; Wimmer et al., 2014) and the head direction system (Redish et al., 1996; Zhang, 1996), among other phenomena. Some models of bump formation have used a firing rate description (Wilson and Cowan, 1973; Amari, 1977; Laing et al., 2002; Laing and Troy, 2003; Owen et al., 2007) while others have considered networks of spiking neurons (Gutkin et al., 2001; Laing and Chow, 2001; Wang, 2001). The simplest models typically have “Mexican-hat” connectivity in a single population of neurons, where nearby neurons are excitatorily coupled and more distant ones are inhibitorily coupled (Ermentrout, 1998; Bressloff, 2012). However, more realistic models consider both excitatory and inhibitory neurons with non-negative connectivity within and between populations (Pinto and Ermentrout, 2001; Blomquist et al., 2005). Almost all previous models have considered homogeneous and isotropic networks, which typically support a continuous family of reflection-symmetric bumps, parameterized by their position in the network. Some exceptions are (Brackley and Turner, 2009, 2014), in which a spatially-inhomogeneous coupling function is used, and (Thul et al., 2016), in which a spatially-varying random firing threshold is imposed.

In this paper we further investigate the effects of breaking the spatial homogeneity of neural networks which support bump solutions, by randomly adding long-range connections and simultaneously removing short-range connections in a particular formulation of small-world networks (Song and Wang, 2014). Small-world networks (Watts and Strogatz, 1998) have been much studied and there is evidence for the existence of small-worldness in several brain networks (Bullmore and Sporns, 2009). In particular, we are interested in determining how sensitive networks which support bumps are to this type of random rewiring of connections, and thus how precisely networks must be constructed in order to support bumps.

We will consider networks of heterogeneous excitatory and inhibitory theta neurons, the theta neuron being the canonical model for a Type I neuron for which the onset of firing is through a saddle-node on an invariant circle bifurcation (Ermentrout and Kopell, 1986; Ermentrout, 1996). In several limits such networks are amenable to the use of the Ott/Antonsen ansatz (Ott and Antonsen, 2008, 2009), and we will build on previous work using this ansatz in the study of networks of heterogeneous theta neurons (Luke et al., 2013; Laing, 2014a, 2015; So et al., 2014). We present the model in Section 2.2 and then consider two limiting cases: an infinite number of neurons (Section 2.3) and an infinite ensemble of finite networks with the same connectivity (Section 2.4). Results are given in Section 3 and we conclude in Section 4. The Appendix contains some mathematical manipulations relating to Section 2.4.

## 2. Materials and methods

### 2.1. Introduction

First consider an all-to-all coupled network of *N* heterogeneous theta neurons whose dynamics are given by

(1) $$\begin{array}{l} \frac{d\theta_{i}}{dt}=1-\text{cos}\theta_{i}+\left(1+\text{cos}\theta_{i}\right)\left(I_{i}+gr\right) \end{array}$$

(2) $$\begin{array}{l} \tau \frac{dr}{dt}=\frac{1}{N}\overset{N}{\underset{j=1}{\sum }}P_{n}\left(\theta_{j}\right)-r \end{array}$$

for *i* = 1, 2, …*N* where θ_*i*_ ∈ [0, 2π) is the phase of the *i*th neuron, $P_{n}\left(\theta \right)=a_{n}{\left(1-\text{cos}\theta \right)}^{n},n\in {\mathbb{N}}^{+}$ and *a*_*n*_ is a normalization factor such that $\int_{0}^{2\pi }P_{n}\left(\theta \right)d\theta =2\pi$. The function *P*_*n*_ is meant to mimic the action potential generated when a neuron fires, i.e., its phase increases through π; *n* controls the “sharpness” of this function. The *I*_*i*_ are input currents randomly chosen from some distribution, *g* is the strength of connectivity within the network (positive for excitatory coupling and negative for inhibitory), and τ is a time constant governing the synaptic dynamics. The variable *r* is driven up by spiking activity and exponentially decays to zero in the absence of activity, on a timescale τ.

The model (1)–(2) with τ = 0 (i.e., instantaneous synapses) was studied by Luke et al. (2013), who found multistability and oscillatory behavior. The case of τ > 0 was considered in Laing, unpublished and similar forms of synaptic dynamics have been considered elsewhere (Börgers and Kopell, 2005; Ermentrout, 2006; Coombes and Byrne, unpublished). The model presented below results from generalizing Equations (1) and (2) in several ways. Firstly, we consider two populations of neurons, one excitatory and one inhibitory. Thus, we will have two sets of variables, one for each population. Such a pair of interacting populations was previously considered by Luke et al. (2014); Börgers and Kopell (2005); Coombes and Byrne, unpublished; and Laing, unpublished. Secondly, we consider a spatially-extended network, in which both the excitatory and inhibitory neurons lie on a ring, and are (initially) coupled to a fixed number of neurons either side of them. Networks with similar structure have been studied by many authors (Redish et al., 1996; Compte et al., 2000; Gutkin et al., 2001; Laing and Chow, 2001; Laing, 2014a, 2015).

### 2.2. Model

We consider a network of 2*N* theta neurons, *N* excitatory and *N* inhibitory. Within each population the neurons are arranged in a ring, and there are synaptic connections between and within populations, whose strength depends on the distance between neurons, as in Laing and Chow (2002) and Gutkin et al. (2001) (In the networks we will consider, connection strengths are either 1 or 0, i.e., neurons are either connected or not connected). Inhibitory synapses act on a timescale τ_*i*_, whereas the excitatory ones act on a timescale, τ. θ_*i*_ ∈ [0, 2π) is the phase of the *i*th excitatory neuron and ϕ_*i*_ ∈ [0, 2π) is the phase of the *i*th inhibitory one. The equations are

(3) $$\begin{array}{l} \frac{d\theta_{i}}{dt}=1-\text{cos}\theta_{i}+\left(1+\text{cos}\theta_{i}\right)\left(I_{i}+g_{EE}v_{i}-g_{EI}y_{i}\right) \end{array}$$

(4) $$\begin{array}{l} \frac{d\phi_{i}}{dt}=1-\text{cos}\phi_{i}+\left(1+\text{cos}\phi_{i}\right)\left(J_{i}+g_{IE}u_{i}-g_{II}z_{i}\right) \end{array}$$

(5) $$\begin{array}{l} \tau \frac{dv_{i}}{dt}=r_{i}-v_{i} \end{array}$$

(6) $$\begin{array}{l} \tau \frac{du_{i}}{dt}=q_{i}-u_{i} \end{array}$$

(7) $$\begin{array}{l} \tau_{i}\frac{dy_{i}}{dt}=s_{i}-y_{i} \end{array}$$

(8) $$\begin{array}{l} \tau_{i}\frac{dz_{i}}{dt}=w_{i}-z_{i} \end{array}$$

for *i* = 1, 2…*N*, where

(9) $$\begin{array}{l} q_{i}=\frac{1}{N}\overset{M_{IE}}{\underset{j=-M_{IE}}{\sum }}P_{n}\left(\theta_{i+j}\right) \end{array}$$

(10) $$\begin{array}{l} r_{i}=\frac{1}{N}\overset{M_{EE}}{\underset{j=-M_{EE}}{\sum }}P_{n}\left(\theta_{i+j}\right) \end{array}$$

(11) $$\begin{array}{l} s_{i}=\frac{1}{N}\overset{M_{EI}}{\underset{j=-M_{EI}}{\sum }}P_{n}\left(\phi_{i+j}\right) \end{array}$$

(12) $$\begin{array}{l} w_{i}=\frac{1}{N}\overset{M_{II}}{\underset{j=-M_{II}}{\sum }}P_{n}\left(\phi_{i+j}\right) \end{array}$$

where *P*_*n*_ is as in Section 2.1. The positive integers *M*_*IE*_, *M*_*EE*_, *M*_*EI*_, and *M*_*II*_ give the width of connectivity from excitatory to inhibitory, excitatory to excitatory, inhibitory to excitatory, and inhibitory to inhibitory populations, respectively. The non-negative quantities *g*_*EE*_, *g*_*EI*_, *g*_*IE*_ and *g*_*II*_ give the overall connection strengths within and between the two populations (excitatory to excitatory, inhibitory to excitatory, excitatory to inhibitory, and inhibitory to inhibitory, respectively). The variable *v*_*i*_ (when multiplied by *g*_*EE*_) gives the excitatory input to the *i*th excitatory neuron, and whose dynamics are driven by *r*_*i*_, which depends on the activity of the excitatory neurons with indices between *i* − *M*_*EE*_ and *i* + *M*_*EE*_. Similarly, *u*_*i*_ (when multiplied by *g*_*IE*_) gives the excitatory input to the *i*th inhibitory neuron, and is driven by *q*_*i*_, which depends on the activity of the excitatory neurons with indices between *i* − *M*_*IE*_ and *i* + *M*_*IE*_. *g*_*EI*_*y*_*i*_ is the inhibitory input to the *i*th excitatory neuron, driven by *s*_*i*_, which depends on the activity of the inhibitory neurons with indices between *i* − *M*_*EI*_ and *i* + *M*_*EI*_. Lastly, *g*_*II*_*z*_*i*_ is the inhibitory input to the *i*th inhibitory neuron, driven by *w*_*i*_, which depends on the activity of the inhibitory neurons with indices between *i* − *M*_*II*_ and *i* + *M*_*II*_.

For simplicity, and motivated by the results in Pinto and Ermentrout (2001), we assume that the inhibitory synapses act instantaneously, i.e. τ_*i*_ = 0, and that there are no connections within the inhibitory population, i.e. *g*_*II*_ = 0. Thus (8) and (12) become irrelevant and from (7) we have that *y*_*i*_ = *s*_*i*_ in (3).

The networks are made heterogeneous by randomly choosing the currents *I*_*i*_ from the Lorentzian

(13) $$\begin{array}{l} h\left(I\right)=\frac{\Delta /\pi }{{\left(I-I_{0}\right)}^{2}+\Delta^{2}} \end{array}$$

and the currents *J*_*i*_ from the Lorentzian

(14) $$\begin{array}{l} g\left(J\right)=\frac{\Delta /\pi }{{\left(J-J_{0}\right)}^{2}+\Delta^{2}}. \end{array}$$

**Figure 1:**
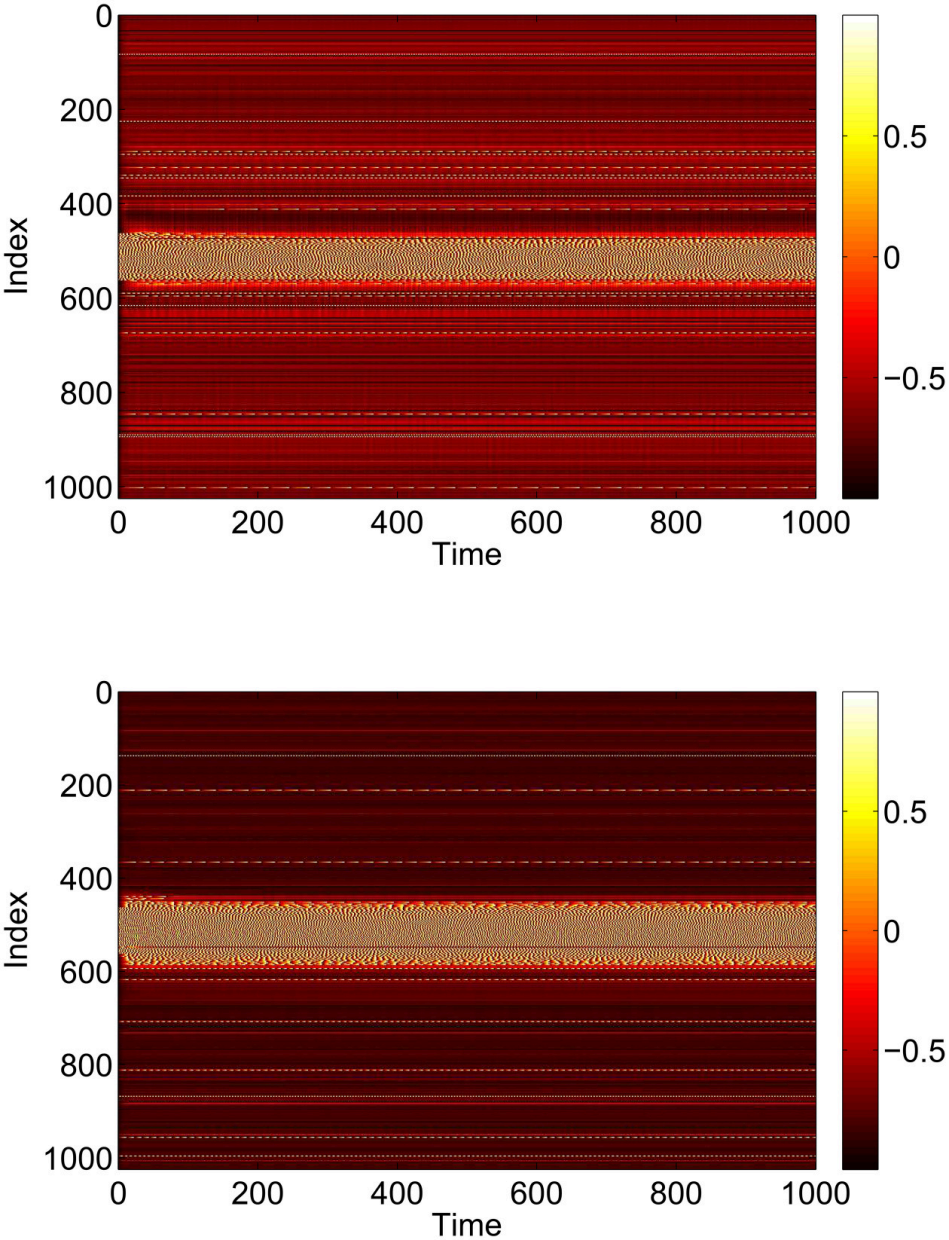
**A bump solution of Equations (3)–(6)**. **Top:** sin θ_*i*_. **Bottom:** sin ϕ_*i*_. Parameter values: *N* = 1024, Δ = 0.02, *I*_0_ = −0.16, *J*_0_ = −0.4, *n* = 2, *g*_*EE*_ = 25, *g*_*IE*_ = 25, *g*_*EI*_ = 7.5, *M*_*IE*_ = 40, *M*_*EE*_ = 40, *M*_*EI*_ = 60 and τ = 10.

**Figure 2:**
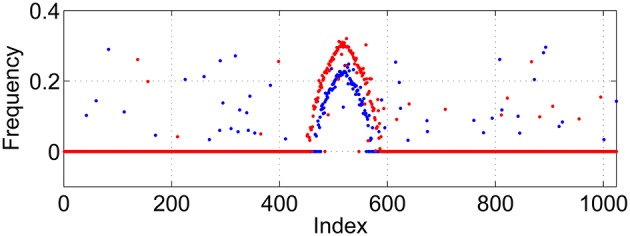
**Average frequency for excitatory population (blue) and inhibitory (red) for the solution shown in Figure 1**.

*I*_0_ and *J*_0_ are the centers of these distributions, and for simplicity we assume that both have the same width, Δ. The heterogeneity of the neurons (i.e., the positive value of Δ) is not necessary in order for the network to support bumps, but it is necessary for the Ott/Antonsen ansatz, used extensively below, to be valid (Ott et al., 2011). Networks of *identical* phase oscillators are known to show non-generic behavior which can be studied using the Watanabe/Strogatz ansatz (Watanabe and Strogatz, 1993, 1994). We want to avoid non-generic behavior, and having a heterogeneous network is also more realistic. For typical parameter values we see the behavior shown in Figures 1, 2, i.e., a stable stationary bump in which the active neurons are spatially localized.

While these bumps may look superficially like “chimera” states in a ring of oscillators (Abrams and Strogatz, 2004, 2006; Laing, 2009; Panaggio and Abrams, 2015) they are different in one important aspect. Chimera states in the references above occur in networks for which the dynamics depend on only *phase differences*. Thus these systems are invariant with respect to adding the same constant to all oscillator phases, and can be studied in a rotating coordinate frame in which the synchronous oscillators have zero frequency, i.e., only *relative* frequencies are meaningful. In contrast, networks of theta neurons like those studied here are *not* invariant with respect to adding the same constant to all oscillator phases. The actual value of phase matters, and the neurons with zero frequency in Figure 2 have zero frequency simply because their input is not large enough to cause them to fire.

**Figure 3:**
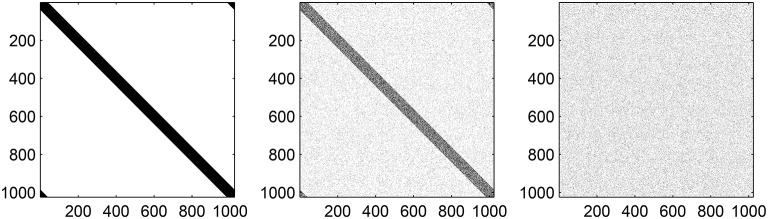
**Typical realizations of ***A***^***IE***^ for ***p***_**1**_ = 0 (left) 0.5 (middle) and 1 (right)**. *N* = 1024, *M*_*IE*_ = 40. Black corresponds to a matrix entry of 1, white to 0.

We now want to introduce rewiring parameters in such a way that on average, the number of connections is preserved as the networks are rewired. This is different from other formulations of small-world networks in which additional edges are added (Newman and Watts, 1999; Medvedev, 2014; but see Puljic and Kozma, 2008 for an example in which the number of connections to a node is precisely conserved). The reason for doing this is to keep the balance of excitation and inhibition constant. If we were to add additional connections, for example, within the excitatory population, the results seen might just be a result of increasing the number of connections, rather than their spatial arrangement. We are interested in the effects of rewiring connections from short range to long range, and thus use the form suggested in Song and Wang (2014). We replace Equations (9) to (11) by

(15) $$\begin{array}{l} q_{i}=\frac{1}{N}\overset{N}{\underset{j=1}{\sum }}A_{ij}^{IE}P_{n}\left(\theta_{j}\right);\;r_{i}=\frac{1}{N}\overset{N}{\underset{j=1}{\sum }}A_{ij}^{EE}P_{n}\left(\theta_{j}\right);\\ s_{i}=\frac{1}{N}\overset{N}{\underset{j=1}{\sum }}A_{ij}^{EI}P_{n}\left(\phi_{j}\right); \end{array}$$

where

(16) $$\begin{array}{l} A_{ij}^{IE}=\{\begin{array}{ll} 1 & \text{with}\\ & \text{probability}\{\begin{array}{ll} 1-[1-(2M_{IE}+1)/N]p_{1}, & |i-j|\leq M_{IE}\\ (2M_{IE}+1)p_{1}/N, & |i-j|>M_{IE} \end{array}\\ 0 & \text{otherwise} \end{array} \end{array}$$

(17) $$\begin{array}{l} A_{ij}^{EE}=\{\begin{array}{ll} 1 & \text{with}\\ & \text{probability}\{\begin{array}{ll} 1-[1-(2M_{EE}+1)/N]p_{2}, & |i-j|\leq M_{EE}\\ (2M_{EE}+1)p_{2}/N, & |i-j|>M_{EE} \end{array}\\ 0 & \text{ otherwise} \end{array} \end{array}$$

and

(18) $$\begin{array}{l} A_{ij}^{EI}=\{\begin{array}{ll} 1 & \text{with}\\ & \text{probability}\{\begin{array}{ll} 1-[1-(2M_{EI}+1)/N]p_{3}, & |i-j|\leq M_{EI}\\ (2M_{EI}+1)p_{3}/N, & |i-j|>M_{EI} \end{array}\\ 0 & \text{ otherwise} \end{array} \end{array}$$

where |*i* − *j*| refers to the shortest distance between neurons *i* and *j*, measured on the ring. When *p*_1_ = *p*_2_ = *p*_3_ = 0, Equation (15) reverts to Equations (9) to (11). Note that when *p*_1_ = 1, the probability of $A_{ij}^{IE}$ being 1 is independent of *i* and *j*, and that the expected number of nonzero entries in a row of $A_{ij}^{IE}$ (i.e., the expected number of connections from the excitatory population to an inhibitory neuron) is independent of *p*_1_. Similar statements apply for the other two matrices and their parameters *p*_2_ and *p*_3_. Typical variation of *A*^*IE*^ with *p*_1_ is shown in Figure 3 and it is clear that increasing *p*_1_ interpolates between purely local connections (*p*_1_ = 0) and uniform random connectivity (*p*_1_ = 1).

We could simply simulate Equations (3)–(6) with Equation (15) for particular values of *p*_1_, *p*_2_, and *p*_3_ but we would like to gain a deeper understanding of the dynamics of such a network. The first approach is to take the continuum limit in which the number of neurons in each network goes to infinity, in a particular way.

### 2.3. Continuum limit

**Figure 4:**
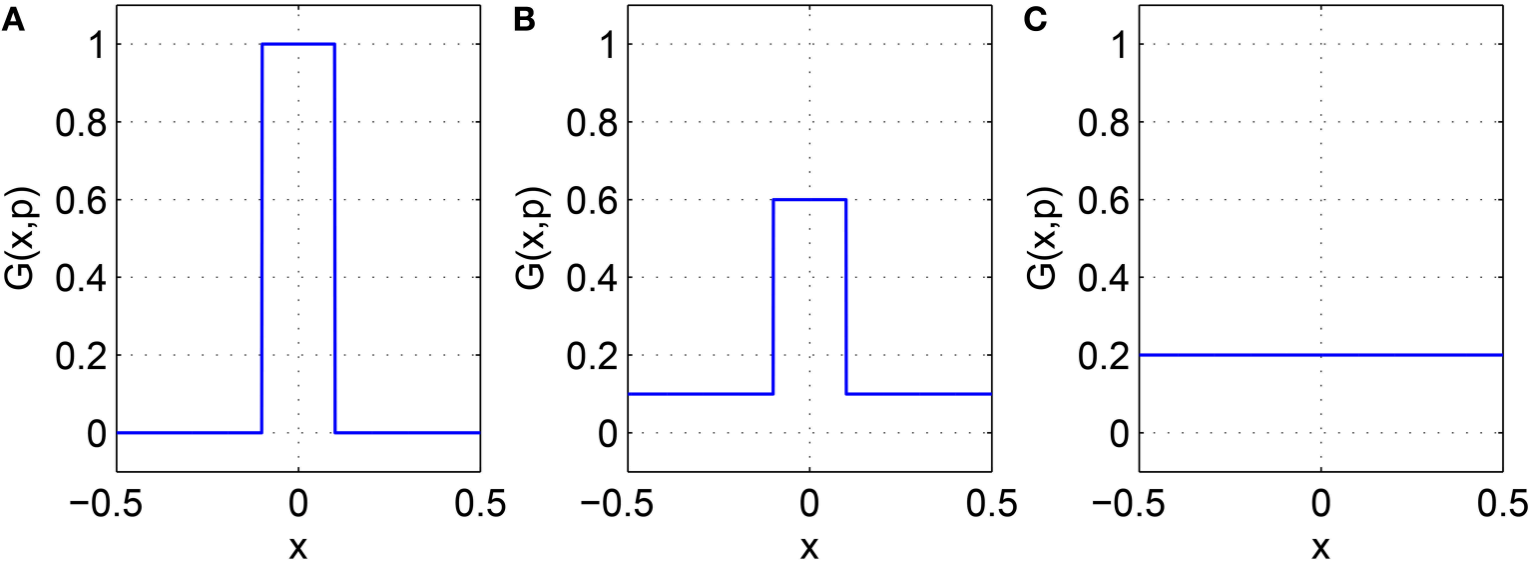
**One of the functions Equations (19)–(21) with (A): ***p*** = 0, (B): ***p*** = 0.5, and (C): ***p*** = 1**. For this example, α = 0.1. Note the similarity with the middle row of the matrices shown in Figure 3.

We take the continuum limit: *N, M*_*EI*_, *M*_*EE*_, *M*_*IE*_ → ∞ such that *M*_*EI*_/*N* → α_*EI*_, *M*_*EE*_/*N* → α_*EE*_ and *M*_*IE*_/*N* → α_*IE*_, where 0 < α_*EI*_, α_*EE*_, α_*IE*_ < 1/2, and set the circumference of the ring of neurons to be 1. In this limit the sums (Equation 15) are replaced by integrals (more specifically, convolutions) with the connectivity kernels

(19) $$\begin{array}{l} G_{IE}(x,p_{1})=\{\begin{array}{ll} 1-(1-2\alpha_{IE})p_{1}, & |x|<\alpha_{IE}\\ 2\alpha_{IE}p_{1}, & \text{otherwise} \end{array} \end{array}$$

(20) $$\begin{array}{l} G_{EE}(x,p_{2})=\{\begin{array}{ll} 1-(1-2\alpha_{EE})p_{2}, & |x|<\alpha_{EE}\\ 2\alpha_{EE}p_{2}, & \text{otherwise} \end{array} \end{array}$$

(21) $$\begin{array}{l} G_{EI}(x,p_{3})=\{\begin{array}{ll} 1-(1-2\alpha_{EI})p_{3}, & |x|<\alpha_{EI}\\ 2\alpha_{EI}p_{3}, & \text{otherwise} \end{array} \end{array}$$

where *G*_*IE*_(*x, p*_1_) is the probability that a point in the excitatory population is connected to a point in the inhibitory population a distance *x* away, and similarly for the other two kernels. The effect of varying *p*_*j*_, *j* = 1, 2, 3, on one of the functions Equations (19)–(21) is shown in Figure 4. Taking *G*_*IE*_ for example, we see that $\int_{-1/2}^{1/2}G_{IE}\left(x,p_{1}\right)\;dx=2\alpha_{IE}$ independent of *p*_1_, i.e., the expected total number of connections is preserved, and similarly for the other two functions.

Taking the continuum limit of Equations (3)–(6) we describe the dynamics of the θ_*i*_ and ϕ_*i*_ in terms of probability densities *F*_*E*_(θ, *x, I, t*) and *F*_*I*_(ϕ, *x, J, t*), respectively, where *x* and *t* are (continuous) space and time, and *I* and *J* are random variables with densities *h*(*I*) and *g*(*J*) respectively. *F*_*E*_ satisfies the continuity equation (Luke et al., 2013)

(22) $$\begin{array}{l} \frac{\partial F_{E}}{\partial t}+\frac{\partial }{\partial \theta }\left\{F_{E}\left[1-\text{cos}\theta +\left(1+\text{cos}\theta \right)\left(I+g_{EE}v-g_{EI}s\right)\right]\right\}=0 \end{array}$$

and similarly *F*_*I*_ satisfies

(23) $$\begin{array}{l} \frac{\partial F_{I}}{\partial t}+\frac{\partial }{\partial \phi }\left\{F_{I}\left[1-\text{cos}\phi +\left(1+\text{cos}\phi \right)\left(J+g_{IE}u\right)\right]\right\}=0 \end{array}$$

where

(24) $$\begin{array}{l} \tau \frac{\partial v}{\partial t}=r-v \end{array}$$

(25) $$\begin{array}{l} \tau \frac{\partial u}{\partial t}=q-u \end{array}$$

and

(26) $$\begin{array}{l} q\left(x,t\right)=\int_{0}^{1}G_{IE}\left(|x-y|,p_{1}\right)\int_{-\infty }^{\infty }\int_{0}^{2\pi }\\ {\;F}_{E}\left(\theta ,y,I,t\right)a_{n}{\left(1-\text{cos}\theta \right)}^{n}d\theta \;dI\;dy \end{array}$$

(27) $$\begin{array}{l} r\left(x,t\right)=\int_{0}^{1}G_{EE}\left(|x-y|,p_{2}\right)\int_{-\infty }^{\infty }\int_{0}^{2\pi }\\ {\;F}_{E}\left(\theta ,y,I,t\right)a_{n}{\left(1-\text{cos}\theta \right)}^{n}d\theta \;dI\;dy \end{array}$$

(28) $$\begin{array}{l} s\left(x,t\right)=\int_{0}^{1}G_{EI}\left(|x-y|,p_{3}\right)\int_{-\infty }^{\infty }\int_{0}^{2\pi }\\ {\;F}_{I}\left(\phi ,y,J,t\right)a_{n}{\left(1-\text{cos}\phi \right)}^{n}d\phi \;dJ\;dy \end{array}$$

The forms of Equations (22) and (23) mean that they are amenable to the use of the Ott/Antonsen ansatz (Ott and Antonsen, 2008, 2009). This ansatz states that if the neurons are not identical (i.e., Δ > 0 for the networks studied here), solutions of the continuity equation [Equations (22) and (23)] decay exponentially onto a lower-dimensional manifold on which the θ and ϕ dependence of *F*_*E*_ and *F*_*I*_, respectively, have a particular form. This form is a Fourier series in θ (or ϕ) in which the *n*th coefficient is some function to the *n*th power. [See Equation (A8), for example]. Thus, we can restrict Equations (22) and (23) to this manifold, thereby simplifying the dynamics.

The standard Kuramoto order parameter for an all-to-all coupled network with phases {θ_*j*_} is the expected value of $e^{i\theta_{j}}$ (Strogatz, 2000). For the network studied here we can define the analogous spatially-dependent order parameters for the excitatory and inhibitory networks as

(29) $$\begin{array}{l} z_{E}\left(x,t\right)=\int_{-\infty }^{\infty }\int_{0}^{2\pi }F_{E}\left(\theta ,x,I,t\right)e^{i\theta }\;d\theta \;dI \end{array}$$

and

(30) $$\begin{array}{l} z_{I}\left(x,t\right)=\int_{-\infty }^{\infty }\int_{0}^{2\pi }F_{I}\left(\phi ,x,J,t\right)e^{i\phi }\;d\phi \;dJ \end{array}$$

respectively. For fixed *x* and *t*, *z*_*E*_(*x, t*) is a complex number with a phase and a magnitude. The phase gives the most likely value of θ and the magnitude governs the “sharpness” of the probability distribution of θ (at that *x* and *t*), and similarly for *z*_*I*_(*x, t*) and ϕ (Laing, 2014a, 2015). We can also determine from *z*_*E*_ and *z*_*I*_ the instantaneous firing rate of each population (see Section 3.1 and Montbrió et al., 2015).

Performing manipulations as in Laing (2014a, 2015), Luke et al. (2013), and So et al. (2014) we obtain the continuum limit of Equations (3)–(6): evolution equations for *z*_*E*_ and *z*_*I*_

(31) $$\begin{array}{l} \frac{\partial z_{E}}{\partial t}=\frac{\left(iI_{0}-\Delta \right){\left(1+z_{E}\right)}^{2}-i{\left(1-z_{E}\right)}^{2}}{2}\\ +\frac{i{\left(1+z_{E}\right)}^{2}\left(g_{EE}v-g_{EI}s\right)}{2} \end{array}$$

(32) $$\begin{array}{l} \frac{\partial z_{I}}{\partial t}=\frac{\left(iJ_{0}-\Delta \right){\left(1+z_{I}\right)}^{2}-i{\left(1-z_{I}\right)}^{2}}{2}+\frac{i{\left(1+z_{I}\right)}^{2}g_{IE}u}{2} \end{array}$$

together with Equations (24) and (25), where

(33) $$\begin{array}{l} q\left(x,t\right)=\int_{0}^{1}G_{IE}\left(|x-y|,p_{1}\right)H\left(z_{E}\left(y,t\right);n\right)dy \end{array}$$

(34) $$\begin{array}{l} r\left(x,t\right)=\int_{0}^{1}G_{EE}\left(|x-y|,p_{2}\right)H\left(z_{E}\left(y,t\right);n\right)dy \end{array}$$

(35) $$\begin{array}{l} s\left(x,t\right)=\int_{0}^{1}G_{EI}\left(|x-y|,p_{3}\right)H\left(z_{I}\left(y,t\right);n\right)dy \end{array}$$

and

(36) $$\begin{array}{l} H\left(z;n\right)=a_{n}\left[C_{0}+\overset{n}{\underset{q=1}{\sum }}C_{q}\left(z^{q}+{\bar{z}}^{q}\right)\right] \end{array}$$

where

(37) $$\begin{array}{l} C_{q}=\overset{n}{\underset{k=0}{\sum }}\overset{k}{\underset{m=0}{\sum }}\frac{n!{\left(-1\right)}^{k}\delta_{k-2m,q}}{2^{k}\left(n-k\right)!m!\left(k-m\right)!} \end{array}$$

and where by |*x* − *y*| in Equations (26)–(28) we mean the shortest distance between *x* and *y* given that they are both points on a circle, i.e., |*x* − *y*| = min (|*x* − *y*|, 1 − |*x* − *y*|).

The advantage of this continuum formulation is that bumps like that in Figure 1 are fixed points of Equations (31) and (32) and Equations (24) and (25). Once these equations have been spatially discretized, we can find fixed points of them using Newton's method, and determine the stability of these fixed points by finding the eigenvalues of the linearization around them. We can also follow these fixed points as parameter are varied, detecting (local) bifurcations (Laing, 2014b). The results of varying *p*_1_, *p*_2_ and *p*_3_ independently are shown in Section 3.1.

### 2.4. Infinite ensembles

We now consider the case where *N* is fixed and finite, and so are the matrices *A*^*IE*^, *A*^*EE*^ and *A*^*EI*^, but we average over an infinite ensemble of networks with these connectivities, where each member of the ensemble has a different (but consistent) realization of the random currents *I*_*i*_ and *J*_*i*_ (Barlev et al., 2011; Laing et al., 2012). This procedure results in 4*N* ordinary differential equations (ODEs), 2*N* of them for complex quantities and the other 2*N* for real quantities. Thus, there is no reduction of dimension from the original system (Equations 3–6), but as in Section 2.3, bump states will be fixed points of these ODEs.

Letting the number of members in the ensemble go to infinity, we describe the state of the excitatory network by the probability density function

(38) $$\begin{array}{l} f^{E}\left(\theta_{1},\theta_{2},\ldots ,\theta_{N};I_{1},I_{2},\ldots I_{N};t\right)\equiv f^{E}\left(\left\{\theta \right\};\left\{I\right\};t\right) \end{array}$$

and that of the inhibitory one by

(39) $$\begin{array}{l} f^{I}\left(\phi_{1},\phi_{2},\ldots ,\phi_{N};J_{1},J_{2},\ldots J_{N};t\right)\equiv f^{I}\left(\left\{\phi \right\};\left\{J\right\};t\right) \end{array}$$

which satisfy the continuity equations

(40) $$\begin{array}{l} \frac{\partial f^{E}}{\partial t}+\overset{N}{\underset{j=1}{\sum }}\frac{\partial }{\partial \theta_{j}}\left[f^{E}\left(\frac{d\theta_{j}}{dt}\right)\right]=0 \end{array}$$

and

(41) $$\begin{array}{l} \frac{\partial f^{I}}{\partial t}+\overset{N}{\underset{j=1}{\sum }}\frac{\partial }{\partial \phi_{j}}\left[f^{I}\left(\frac{d\phi_{j}}{dt}\right)\right]=0 \end{array}$$

where *dθ*_*j*_/*dt* and *dϕ*_*j*_/*dt* are given by Equations (3) and (4).

Performing the manipulations in the Appendix we obtain

(42) $$\begin{array}{l} \frac{dz_{j}^{E}}{dt}=\frac{\left(iI_{0}-\Delta \right){\left(1+z_{j}^{E}\right)}^{2}-i{\left(1-z_{j}^{E}\right)}^{2}}{2}\\ \;+\frac{i{\left(1+z_{j}^{E}\right)}^{2}\left(g_{EE}v_{j}-g_{EI}s_{j}\right)}{2} \end{array}$$

(43) $$\begin{array}{l} \frac{dz_{j}^{I}}{dt}=\frac{\left(iJ_{0}-\Delta \right){\left(1+z_{j}^{I}\right)}^{2}-i{\left(1-z_{j}^{I}\right)}^{2}}{2}+\frac{i{\left(1+z_{j}^{I}\right)}^{2}g_{IE}u_{j}}{2} \end{array}$$

for *j* = 1, 2, …*N* where

(44) $$\begin{array}{l} q_{i}=\frac{1}{N}\overset{N}{\underset{j=1}{\sum }}A_{ij}^{IE}H\left(z_{j}^{E}\left(t\right);n\right) \end{array}$$

(45) $$\begin{array}{l} r_{i}=\frac{1}{N}\overset{N}{\underset{j=1}{\sum }}A_{ij}^{EE}H\left(z_{j}^{E}\left(t\right);n\right) \end{array}$$

(46) $$\begin{array}{l} s_{i}=\frac{1}{N}\overset{N}{\underset{j=1}{\sum }}A_{ij}^{EI}H\left(z_{j}^{I}\left(t\right);n\right) \end{array}$$

and

(47) $$\begin{array}{l} \tau \frac{dv_{i}}{dt}=r_{i}-v_{i} \end{array}$$

(48) $$\begin{array}{l} \tau \frac{du_{i}}{dt}=q_{i}-u_{i} \end{array}$$

for *i* = 1, 2, …*N*. Equations (42)–(48) form a complete description of the *expected* behavior of a network with connectivities given by the matrices *A*^*IE*^, *A*^*EE*^ and *A*^*EI*^. Note the similarities with Equations (31)–(35) and Equations (24) and (25). As mentioned above, the advantage of this formulation is that states like that in Figure 1 will be fixed points of Equations (42)–(48), for the specified connectivities.

Recalling that the matrices *A*^*IE*^, *A*^*EE*^ and *A*^*EI*^ depend on the parameters *p*_1_, *p*_2_ and *p*_3_ respectively we now investigate how solutions of Equations (42)–(48) depend on these parameters. One difficulty in trying to vary, say, *p*_1_, is that the entries of *A*^*IE*^ do not depend continuously on *p*_1_. Indeed, as presented, one should recalculate *A*^*IE*^ each time *p*_1_ is changed. In order to generate results comparable with those from Section 2.3 we introduce a consistent family of matrices, following Medvedev (2014). Consider *A*^*IE*^ (similar procedures apply for the other two matrices) and define an *N* × *N* matrix *r*, each entry of which is independently and randomly chosen from a uniform distribution on the interval (0, 1). The matrix *r* is now considered to be fixed, and we define $A^{IE}\left(p_{1}\right)$ as follows:

(49) $$\begin{array}{l} A_{ij}^{IE}(p_{1})=\{\begin{array}{cc} \Theta [r_{ij}-p_{1}(1-(2M_{IE}+1)/N)], & |i-j|\leq M_{IE}\\ \Theta [r_{ij}-(1-p_{1}(2M_{IE}+1)/N)], & |i-j|>M_{IE} \end{array} \end{array}$$

where Θ is the Heaviside step function and the indices are taken modulo *N*. Comparing this with Equation (16) we see that for a fixed *p*_1_, generating a new *r* and using Equation (49) is equivalent to generating *A*^*IE*^ using Equation (16). The reason for using Equation (49) is that since the *r*_*ij*_ are chosen once and then fixed, an entry in *A*^*IE*^ will switch from 0 to 1 (or vice versa) at most once as *p*_1_ is varied monotonically in the interval [0, 1].

The effects of quasistatically increasing *p*_1_ and *p*_3_ for Equations (42)–(48) are shown in Section 3.2.

## 3. Results

### 3.1. Results for continuum limit

**Figure 5:**
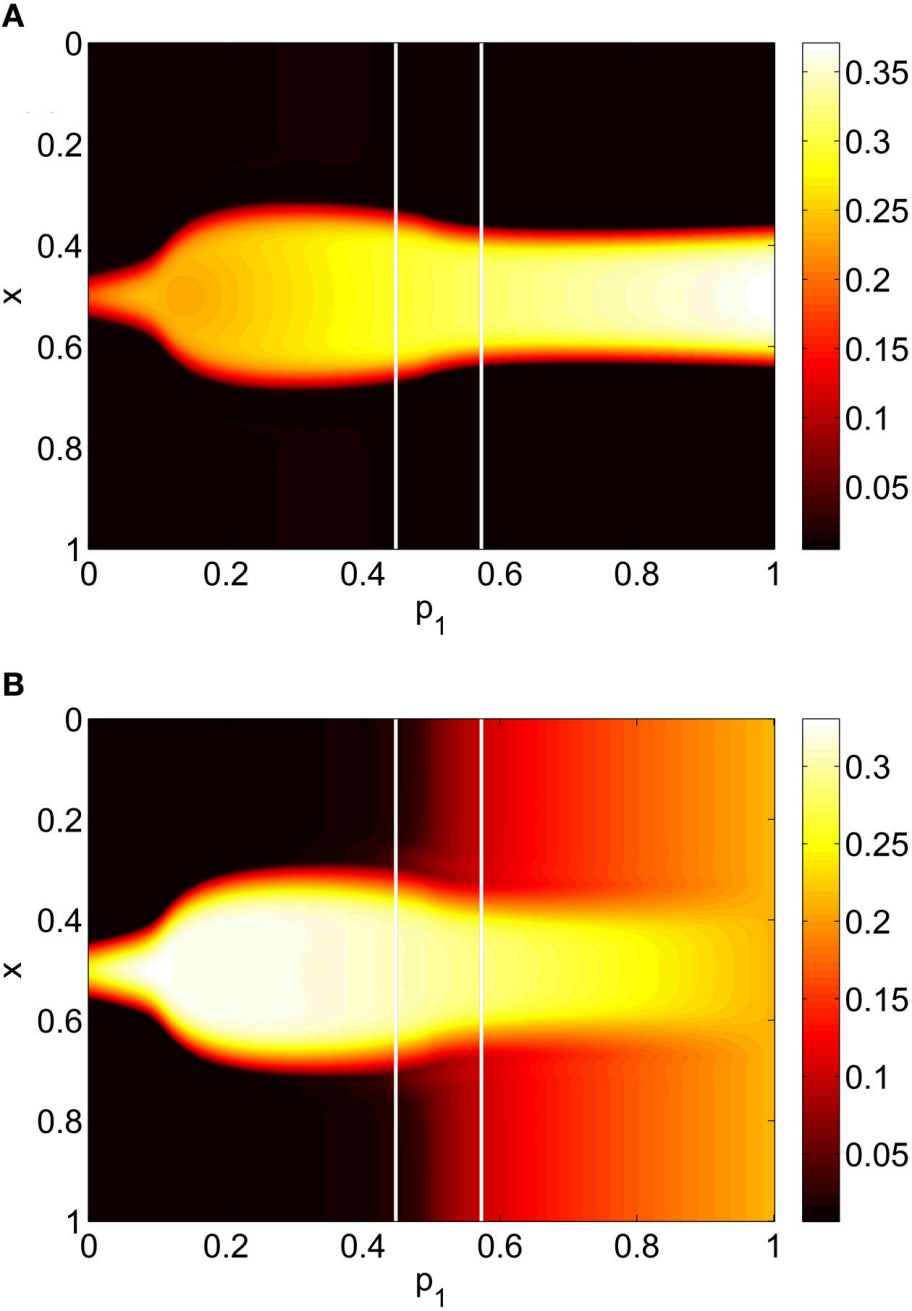
**Firing rate for (A): excitatory population and (B): inhibitory population, as a function of ***p***_**1**_, with ***p***_**2**_ = ***p***_**3**_ = 0**. There is a Hopf bifurcation on both white vertical lines and the bump is unstable between these. Other parameters: Δ = 0.02, *I*_0_ = −0.16, *J*_0_ = −0.4, *n* = 2, *g*_*EE*_ = 25, *g*_*IE*_ = 25, *g*_*EI*_ = 7.5, α_*IE*_ = 40/1024, α_*EE*_ = 40/1024, α_*EI*_ = 60/1024 and τ = 10.

For the system Equations (31) and (32) and Equations (24) and (25) we discretize the spatial domain into 1024 evenly spaced points and approximate the integrals in Equations (33)–(35) with Riemann sums. We numerically integrate the spatially-discretized evolution equations in time, using appropriate initial conditions, until a steady state is reached. This steady state is then continued using pseudo-arclength continuation, and the stability of the solutions found determined by examining the eigenvalues of the Jacobian evaluated at them (Laing, 2014b). The increment between successive values of the *p*_*i*_ found during continuation is not fixed and the numerical results found were interpolated to a uniform grid for plotting in Figures 5, **7**, **8**. We consider varying *p*_1_, *p*_2_ and *p*_3_ independently, keeping the other two parameters fixed at zero. The results of varying *p*_1_ are shown in Figure 5, where we plot the firing rate of the two populations, derived as Re(*w*_*i*_)/π where $w_{i}=\left(1-{\bar{z}}_{i}\right)/\left(1+{\bar{z}}_{i}\right)$ for *i* = *I, E*, as in Montbrió et al. (2015), where the *z*_*i*_ are fixed points of Equations (31) and (32). We see an increase and then decrease in bump width as *p*_1_ is increased. There is also a pair of supercritical Hopf bifurcations, between which the bump is unstable (It is only weakly unstable, with the rightmost eigenvalue of the Jacobian having a maximal real part of 0.015 in this interval). At the leftmost Hopf bifurcation the Jacobian has eigenvalues ±1.8191*i* and at the rightmost it has eigenvalues ±1.7972*i*, with all other eigenvalues having negative real parts. One notable aspect is the increase in firing rate of the inhibitory population “outside” the bump as *p*_1_ is increased, such that when *p*_1_ = 1 the firing rate in this population is spatially homogeneous. This is to be expected, as there are no inhibitory-to-inhibitory connections, and when *p*_1_ = 1 all inhibitory neurons receive the same input from the excitatory population.

**Figure 6:**
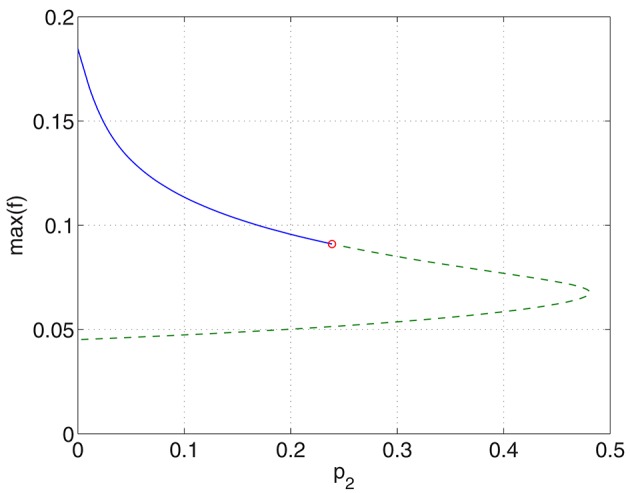
**Maximum (over ***x***) of the firing rate for the excitatory population as a function of ***p***_**2**_ with ***p***_**1**_ = ***p***_**3**_ = 0**. Solid: stable; dashed: unstable. The Hopf bifurcation is marked with a circle. Other parameters: Δ = 0.02, *I*_0_ = −0.16, *J*_0_ = −0.4, *n* = 2, *g*_*EE*_ = 25, *g*_*IE*_ = 25, *g*_*EI*_ = 7.5, α_*IE*_ = 40/1024, α_*EE*_ = 40/1024, α_*EI*_ = 60/1024 and τ = 10.

**Figure 7:**
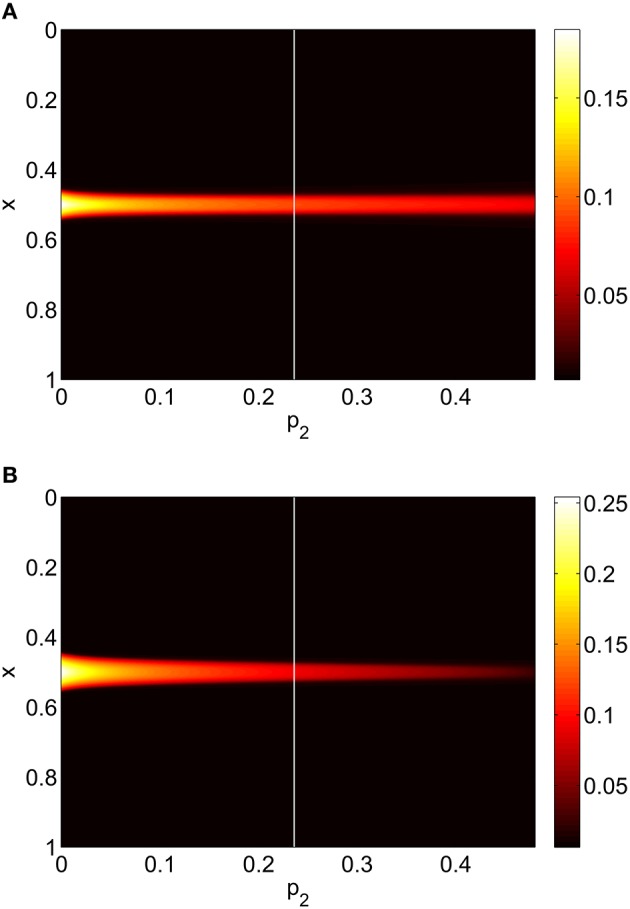
**Firing rate for (A): excitatory population and (B): inhibitory population, as a function of ***p***_**2**_, with ***p***_**3**_ = ***p***_**1**_ = 0**. There is a Hopf bifurcation at the white vertical line and the bump is destroyed in saddle-node bifurcation at *p*_2_ ≈ 0.48. Other parameters: Δ = 0.02, *I*_0_ = −0.16, *J*_0_ = −0.4, *n* = 2, *g*_*EE*_ = 25, *g*_*IE*_ = 25, *g*_*EI*_ = 7.5, α_*IE*_ = 40/1024, α_*EE*_ = 40/1024, α_*EI*_ = 60/1024 and τ = 10.

Increasing *p*_2_ while keeping *p*_1_ = *p*_3_ = 0 we find that the bump undergoes a Hopf bifurcation (Jacobian has eigenvalues ±0.3404*i*) and then is destroyed in a saddle-node bifurcation at *p*_2_ ≈ 0.48, as shown in Figure 6. The behavior of the bumps for 0 ≤ *p*_2_ ≤ 0.48 is shown in Figure 7.

**Figure 8:**
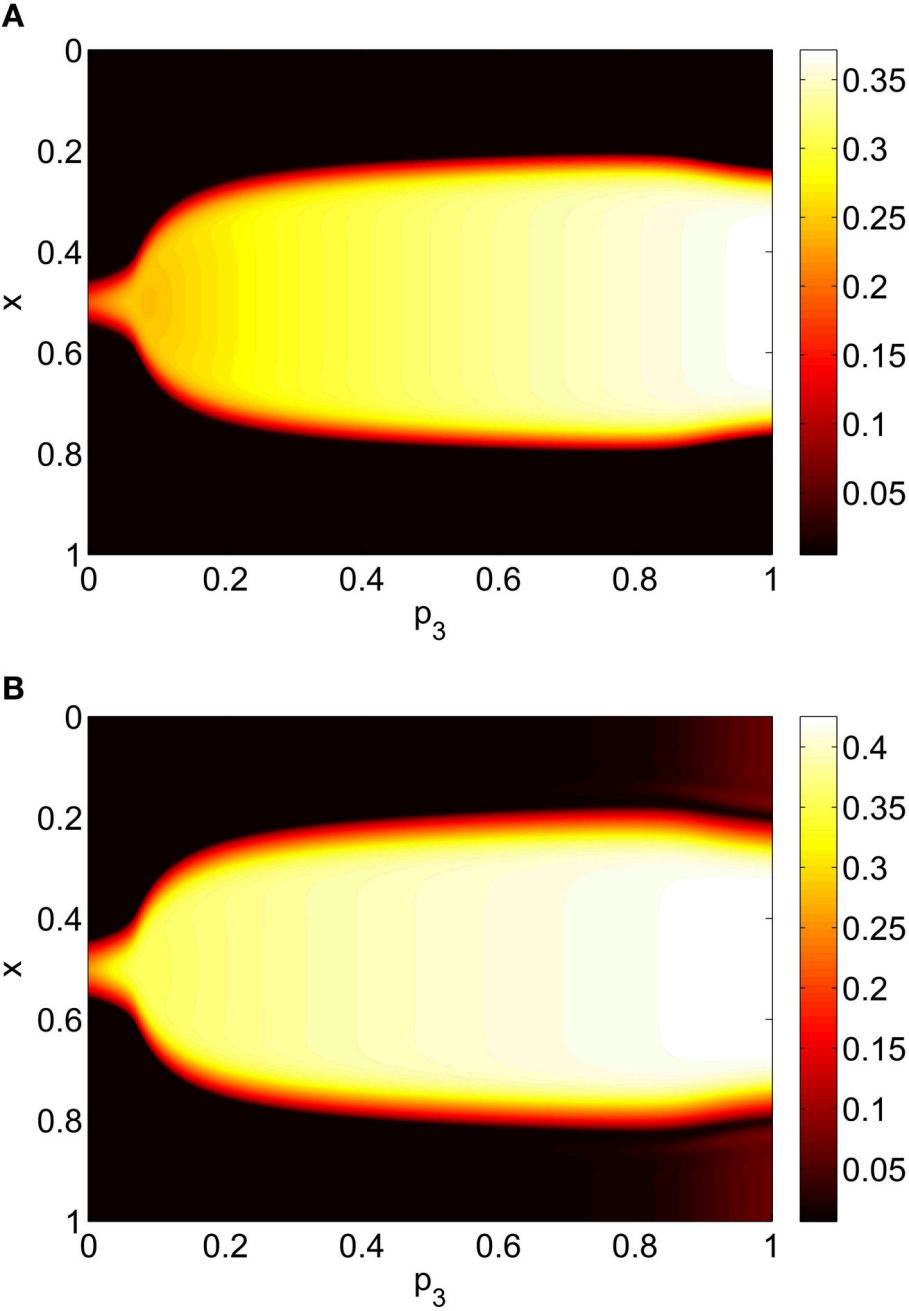
**Firing rate for (A): excitatory population and (B): inhibitory population, as a function of ***p***_**3**_, with ***p***_**2**_ = ***p***_**1**_ = 0**. Other parameters: Δ = 0.02, *I*_0_ = −0.16, *J*_0_ = −0.4, *n* = 2, *g*_*EE*_ = 25, *g*_*IE*_ = 25, *g*_*EI*_ = 7.5, α_*IE*_ = 40/1024, α_*EE*_ = 40/1024, α_*EI*_ = 60/1024 and τ = 10.

Varying *p*_3_ we obtain Figure 8, where there are no bifurcations as *p*_3_ is increased all the way to 1, corresponding to the case where all excitatory neurons feel the same inhibition, just a weighted mean of the output from the inhibitory population. We again see an increase and then slight decrease in bump width as *p*_3_ is increased.

While a Hopf bifurcation of a bump may seem undesirable from a neurocomputational point of view, it should be kept in mind that oscillations are an essential phenomenon in many different neural networks, and they are widely studied (Ashwin et al., 2016).

We have only varied one of *p*_1_, *p*_2_ and *p*_3_, keeping the other two probabilities at zero. A clearer picture of the system's behavior could be obtained by simultaneously varying two, or all three, of these probabilities. We leave this as future work, but mention that for the special case *p*_1_ = *p*_2_ = *p*_3_ = *p*, the bump persists and is stable up to *p* ≈ 0.49, where it undergoes a saddle-node bifurcation (not shown).

### 3.2. Results for infinite ensemble

**Figure 9:**
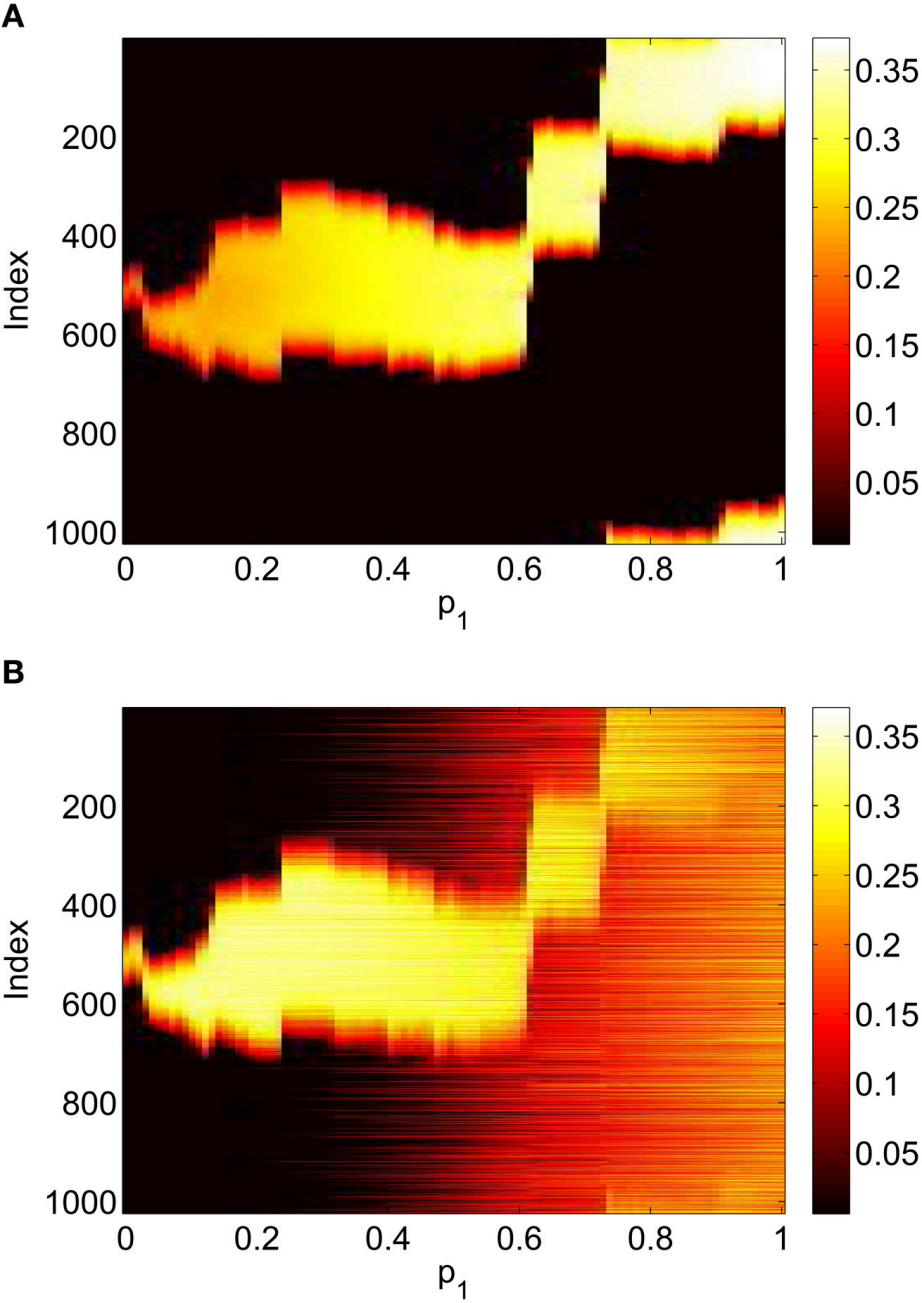
**Firing rate for (A): excitatory population and (B): inhibitory population, as a function of ***p***_**1**_, with ***p***_**2**_ = ***p***_**3**_ = 0**. Compare with Figure 5. Other parameters: Δ = 0.02, *I*_0_ = −0.16, *J*_0_ = −0.4, *n* = 2, *g*_*EE*_ = 25, *g*_*IE*_ = 25, *g*_*EI*_ = 7.5, *N* = 1024, *M*_*IE*_ = 40, *M*_*EE*_ = 40, *M*_*EI*_ = 60 and τ = 10.

This section refers to Equations (42)–(48). In Figure 9 we show the results of slowly increasing *p*_1_, while keeping *p*_2_ = *p*_3_ = 0. We initially set *p*_1_ = 0 and integrated Equations (42)–(48) to a steady state, using initial conditions that give a bump solution. We then increased *p*_1_ by 0.01 and integrated Equations (42)–(48) again for 10,000 time units, using as an initial condition the final state of the previous integration. We continued this process up to *p*_1_ = 1. The firing rate for the *j*th excitatory neuron is Re(*w*_*j*_)/π where $w_{j}=\left(1-{\bar{z}}_{j}^{E}\right)/\left(1+{\bar{z}}_{j}^{E}\right)$, and similarly for an inhibitory neuron. Comparing Figure 9 with Figure 5 we see the same behavior, the main difference being that the bump now moves in an unpredictable way around the domain as *p*_1_ is increased. This is due to the system no longer being translationally invariant, and the bump moving to a position in which it is stable (Thul et al., 2016). Unlike the situation shown in Figure 5 we did not observe any Hopf bifurcations, for this realization of the *A*^*IE*^. Presumably this is also a result of breaking the translational invariance and the weakly unstable nature of the bump shown in Figure 5 between the Hopf bifurcations.

**Figure 10:**
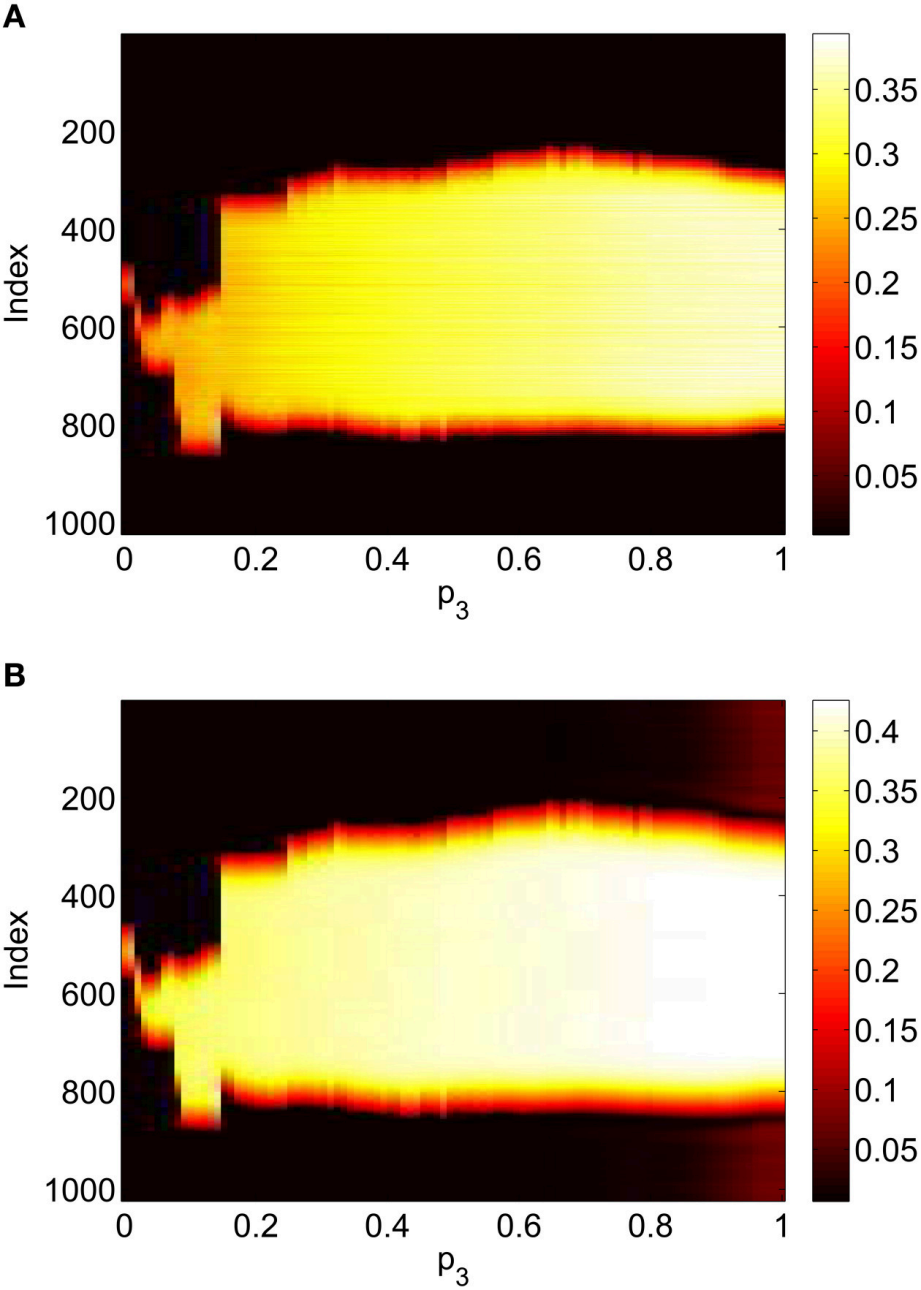
**Firing rate for (A): excitatory population and (B): inhibitory population, as a function of ***p***_**3**_, with ***p***_**2**_ = ***p***_**1**_ = 0**. Compare with Figure 8. Other parameters: Δ = 0.02, *I*_0_ = −0.16, *J*_0_ = −0.4, *n* = 2, *g*_*EE*_ = 25, *g*_*IE*_ = 25, *g*_*EI*_ = 7.5, *N* = 1024, *M*_*IE*_ = 40, *M*_*EE*_ = 40, *M*_*EI*_ = 60 and τ = 10.

**Figure 11:**
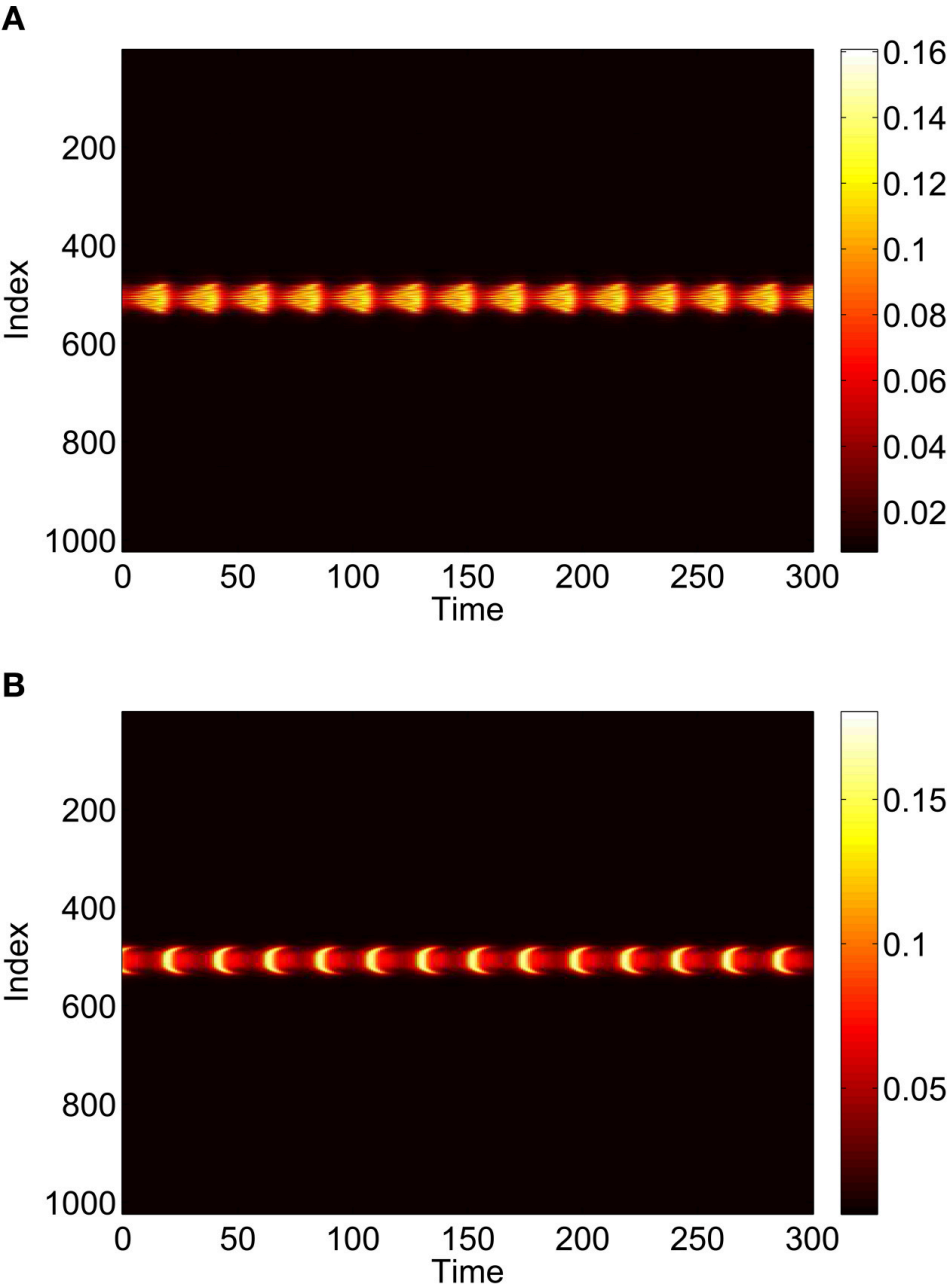
**Instantaneous firing rate for (A): excitatory population and (B): inhibitory population, with ***p***_**2**_ = 0.3 and ***p***_**3**_ = ***p***_**1**_ = 0**. Other parameters: Δ = 0.02, *I*_0_ = −0.16, *J*_0_ = −0.4, *n* = 2, *g*_*EE*_ = 25, *g*_*IE*_ = 25, *g*_*EI*_ = 7.5, *N* = 1024, *M*_*IE*_ = 40, *M*_*EE*_ = 40, *M*_*EI*_ = 60 and τ = 10.

Repeating this process as *p*_3_ is varied with *p*_1_ = *p*_2_ = 0 we obtain the results in Figure 10. Comparing with Figure 8 we see very good agreement, although the bump does move considerably for small *p*_3_, as in Figure 9. Varing *p*_2_ with *p*_1_ = *p*_3_ = 0 we obtain similar results to those in Figure 7 (not shown). Typical behavior of (42)–(48) with *p*_2_ = 0.3 (i.e., beyond the Hopf bifurcation shown in Figure 7) is shown in Figure 11, where the oscillations are clearly seen.

### 3.3. Results for original network

**Figure 12:**
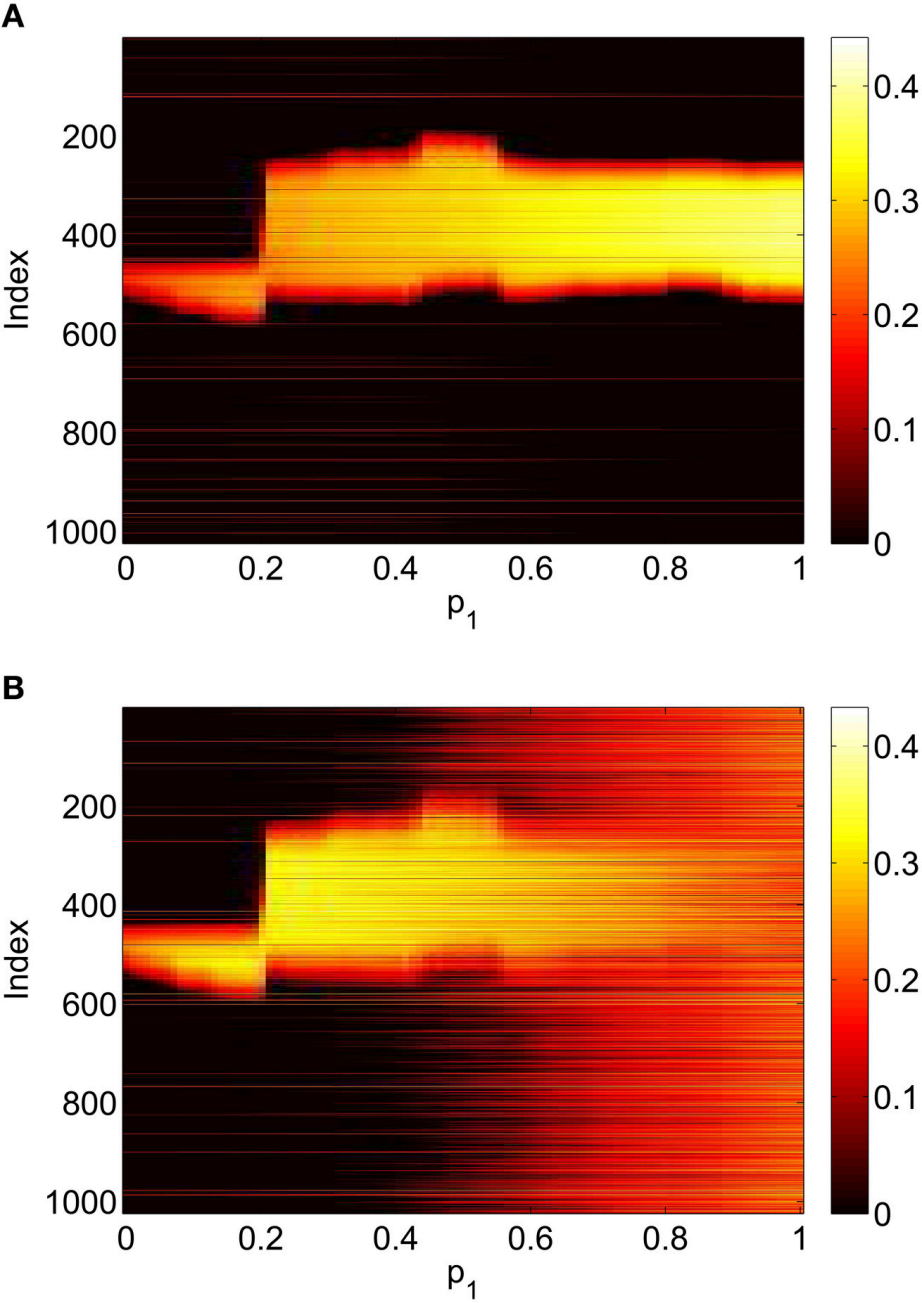
**Firing rate for (A): excitatory population and (B): inhibitory population in the full network Equations (3)–(6), averaged over a time window of length 500, as a function of ***p***_**1**_ with ***p***_**2**_ = ***p***_**3**_ = 0**. Compare with Figure 9. Other parameters: Δ = 0.02, *I*_0_ = −0.16, *J*_0_ = −0.4, *n* = 2, *g*_*EE*_ = 25, *g*_*IE*_ = 25, *g*_*EI*_ = 7.5, *N* = 1024, *M*_*IE*_ = 40, *M*_*EE*_ = 40, *M*_*EI*_ = 60 and τ = 10.

**Figure 13:**
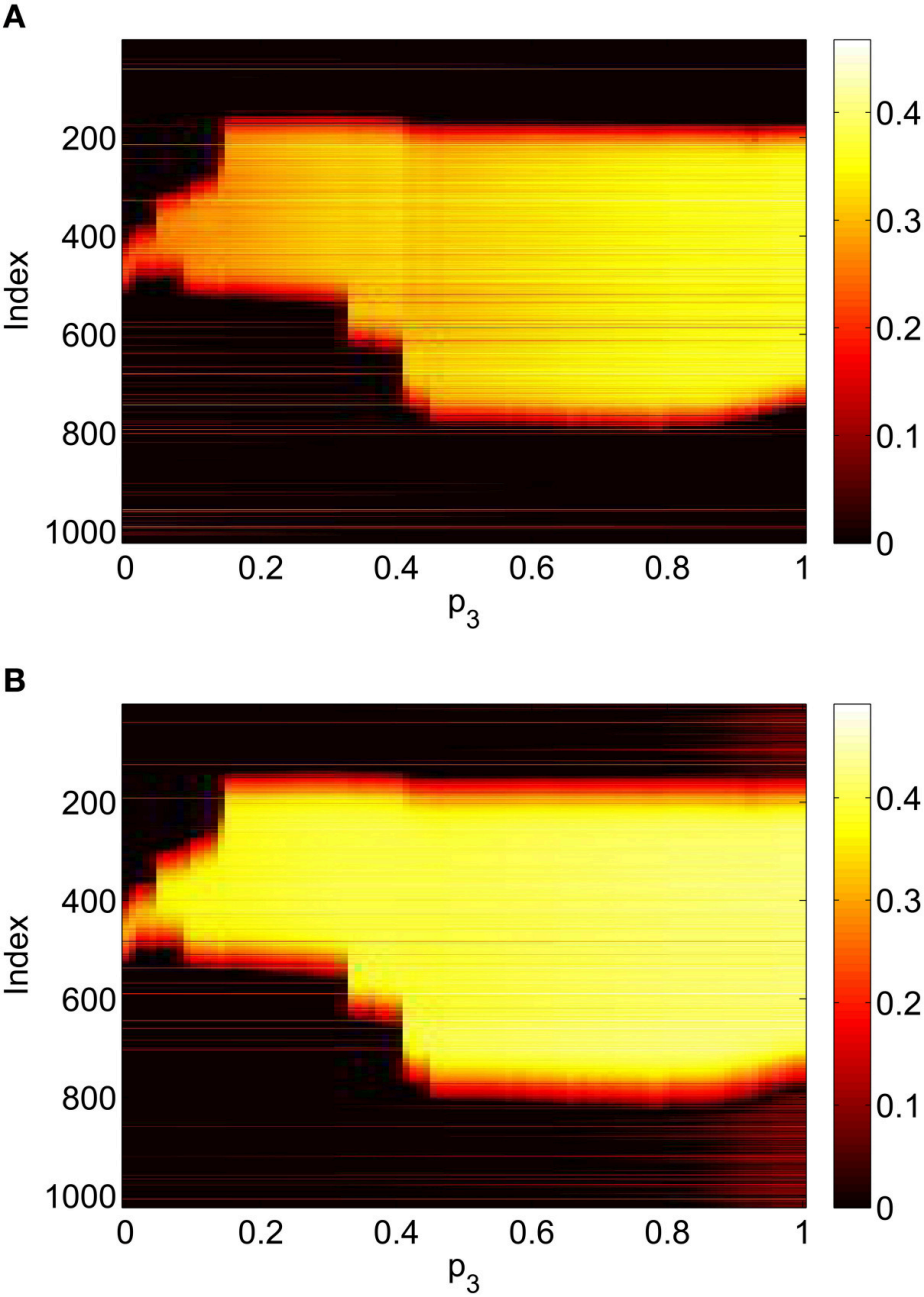
**Firing rate for (A): excitatory population and (B): inhibitory population in the full network Equations (3)–(6), averaged over a time window of length 500, as a function of ***p***_**3**_ with ***p***_**2**_ = ***p***_**1**_ = 0**. Compare with Figure 10. Other parameters: Δ = 0.02, *I*_0_ = −0.16, *J*_0_ = −0.4, *n* = 2, *g*_*EE*_ = 25, *g*_*IE*_ = 25, *g*_*EI*_ = 7.5, *N* = 1024, *M*_*IE*_ = 40, *M*_*EE*_ = 40, *M*_*EI*_ = 60 and τ = 10.

**Figure 14:**
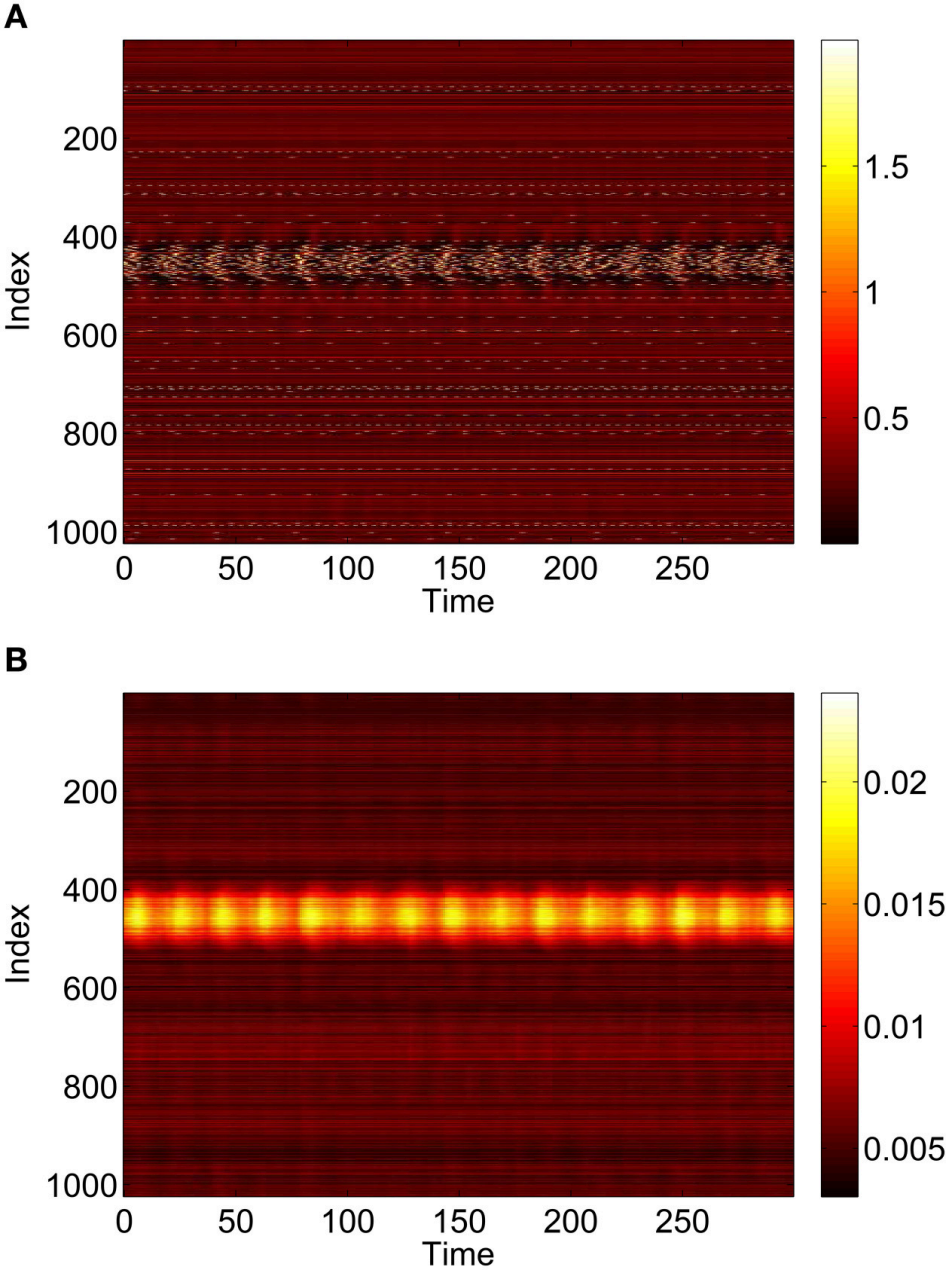
**Behavior of the full network Equations (3)–(6) with ***p***_**2**_ = 0.3 and ***p***_**3**_ = ***p***_**1**_ = 0**. **(A)**: 1 − cos θ_*j*_, **(B)**: *v*_*j*_. Compare with Figure 11. Other parameters: Δ = 0.02, *I*_0_ = −0.16, *J*_0_ = −0.4, *n* = 2, *g*_*EE*_ = 25, *g*_*IE*_ = 25, *g*_*EI*_ = 7.5, *N* = 1024, *M*_*IE*_ = 40, *M*_*EE*_ = 40, *M*_*EI*_ = 60 and τ = 10.

To verify the results obtained above we ran the full network Equations (3)–(6) but using the connectivity Equation (49) (and similar constructions for *A*^*EE*^ and *A*^*EI*^) to calculate Equation (15). The frequency was measured directly from simulations. Varying *p*_1_ we obtain the results in Figure 12; again, no oscillatory behavior associated with a Hopf bifurcation was observed and the results are similar to those in Figure 9. Varying *p*_3_ we obtained Figure 13 (compare with Figure 10). Figure 14 shows oscillatory behavior at *p*_2_ = 0.3, *p*_1_ = *p*_3_ = 0 (the same parameter values as used in Figure 11).

Note that the results in Figures 9–14 are each for a single realization of a typical (parameterized) small-world network. To gain insight into general small-world networks it would be of interest to study the statistics of the behavior of such networks.

## 4. Discussion

We have considered the effects of randomly adding long-range and simultaneously removing short-range connections in a network of model theta neurons which is capable of supporting spatially localized bump solutions. Such rewiring makes the networks small-world, at least for small values of the rewiring probabilities. By using theta neurons we are able to use the Ott/Antonsen ansatz to derive descriptions of the networks in two limits: an infinite number of neurons, and an infinite ensemble of finite networks, each with the same connectivity. The usefulness of this is that the bumps of interest are *fixed points* of the dynamical equations derived in these ways, and can thus be found, their stability determined, and followed as parameters are varied using standard dynamical systems techniques.

For the parameters chosen we found bumps to be surprisingly robust: in several cases a rewiring probability could be taken from 0 to 1 without destroying a bump. However, rewiring connections within the excitatory population (increasing *p*_2_) was found to destabilize a bump through a Hopf bifurcation and later destroy the unstable bump in a saddle-node bifurcation. Simulations of the full network were used to verify our results.

The network studied has many parameters: the spatial spread of local couplings, the timescale of excitatory synapses, the connection strengths within and between populations, and the distributions of heterogeneous input currents. These were all set so that the network without rewiring supported a stable bump solution, but we have not investigated the effects of varying any of these parameters. However, even without considering rewiring, Equations (31)–(35) and Equations (24) and (25) provide a framework for investigating the effects of varying these parameters on the existence and stability of bump solutions, since these continuum equations are derived directly from networks of spiking neurons, unlike many neural field models.

## Author contributions

All authors listed, have made substantial, direct and intellectual contribution to the work, and approved it for publication.

### Conflict of interest statement

The author declares that the research was conducted in the absence of any commercial or financial relationships that could be construed as a potential conflict of interest.

## Appendix

### Mathematical details relating to section 2.4

In the limit of an infinite ensemble we have

(A1) $$\begin{array}{l} q_{i}=\frac{1}{N}\overset{N}{\underset{j=1}{\sum }}A_{ij}^{IE}\int \cdot \cdot \cdot \int P_{n}\left(\theta_{j}\right)f^{E}\left(\left\{\theta \right\};\left\{I\right\};t\right)\;d\theta_{1}\;d\theta_{2}\ldots d\theta_{N}\;dI_{1}\;dI_{2}\ldots dI_{N}\\ \;=\frac{1}{N}\overset{N}{\underset{j=1}{\sum }}A_{ij}^{IE}\int_{-\infty }^{\infty }\int_{0}^{2\pi }P_{n}\left(\theta_{j}\right)f_{j}^{E}\left(\theta_{j},I_{j},t\right)d\theta_{j}\;dI_{j} \end{array}$$

(A2) $$\begin{array}{l} r_{i}=\frac{1}{N}\overset{N}{\underset{j=1}{\sum }}A_{ij}^{EE}\int \cdot \cdot \cdot \int P_{n}\left(\theta_{j}\right)f^{E}\left(\left\{\theta \right\};\left\{I\right\};t\right)\;d\theta_{1}\;d\theta_{2}\ldots d\theta_{N}\;dI_{1}\;dI_{2}\ldots dI_{N}\\ \;=\frac{1}{N}\overset{N}{\underset{j=1}{\sum }}A_{ij}^{EE}\int_{-\infty }^{\infty }\int_{0}^{2\pi }P_{n}\left(\theta_{j}\right)f_{j}^{E}\left(\theta_{j},I_{j},t\right)d\theta_{j}\;dI_{j} \end{array}$$

(A3) $$\begin{array}{l} s_{i}=\frac{1}{N}\overset{N}{\underset{j=1}{\sum }}A_{ij}^{EI}\int \cdot \cdot \cdot \int P_{n}\left(\phi_{j}\right)f^{I}\left(\left\{\phi \right\};\left\{J\right\};t\right)\;d\phi_{1}\;d\phi_{2}\ldots d\phi_{N}\;dJ_{1}\;dJ_{2}\ldots dJ_{N}\\ \;=\frac{1}{N}\overset{N}{\underset{j=1}{\sum }}A_{ij}^{EI}\int_{-\infty }^{\infty }\int_{0}^{2\pi }P_{n}\left(\phi_{j}\right)f_{j}^{I}\left(\phi_{j},J_{j},t\right)d\phi_{j}\;dJ_{j} \end{array}$$

where $f_{j}^{E}\left(\theta_{j},I_{j},t\right)$ is the marginal distribution for θ_*j*_, given by

(A4) $$\begin{array}{l} f_{j}^{E}\left(\theta_{j},I_{j},t\right)=\int \cdot \cdot \cdot \int f^{E}\left(\left\{\theta \right\};\left\{I\right\};t\right)\underset{k\neq j}{\prod }d\theta_{k}\;dI_{k} \end{array}$$

and similarly

(A5) $$\begin{array}{l} f_{j}^{I}\left(\phi_{j},J_{j},t\right)=\int \cdot \cdot \cdot \int f^{I}\left(\left\{\phi \right\};\left\{J\right\};t\right)\underset{k\neq j}{\prod }d\phi_{k}\;dJ_{k} \end{array}$$

Multiplying the continuity equation (40) by ∏_*k*≠*j*_ *dθ*_*k*_ *dI*_*k*_ and integrating we find that each $f_{j}^{E}$ satisfies

(A6) $$\begin{array}{l} \frac{\partial f_{j}^{E}}{\partial t}+\frac{\partial }{\partial \theta_{j}}\left[f_{j}^{E}\left(\frac{d\theta_{j}}{dt}\right)\right]=0 \end{array}$$

Similarly each $f_{j}^{I}$ satisfies

(A7) $$\begin{array}{l} \frac{\partial f_{j}^{I}}{\partial t}+\frac{\partial }{\partial \phi_{j}}\left[f_{j}^{I}\left(\frac{d\phi_{j}}{dt}\right)\right]=0 \end{array}$$

Using the Ott/Antonsen ansatz we write

(A8) $$\begin{array}{l} f_{j}^{E}\left(\theta_{j},I_{j},t\right)=\frac{h\left(I_{j}\right)}{2\pi }\left\{1+\overset{\infty }{\underset{n=1}{\sum }}{\left[\alpha_{j}^{E}\left(I_{j},t\right)\right]}^{n}e^{in\theta_{j}}+c.c.\right\} \end{array}$$

and

(A9) $$\begin{array}{l} f_{j}^{I}\left(\phi_{j},J_{j},t\right)=\frac{g\left(J_{j}\right)}{2\pi }\left\{1+\overset{\infty }{\underset{n=1}{\sum }}{\left[\alpha_{j}^{I}\left(J_{j},t\right)\right]}^{n}e^{in\phi_{j}}+c.c.\right\} \end{array}$$

for some functions $\alpha_{j}^{E}\left(I_{j},t\right)$ and $\alpha_{j}^{I}\left(J_{j},t\right)$, where “*c*.*c*.” means the complex conjugate of the previous term. Substituting Equation (A8) into Equation (A6) and Equation (A9) into Equation (A7) we find that

(A10) $$\begin{array}{l} \frac{\partial \alpha_{j}^{E}}{\partial t}=-i\left[\frac{I_{j}+g_{EE}v_{j}-g_{EI}s_{j}-1}{2}+\left(I_{j}+g_{EE}v_{j}-g_{EI}s_{j}+1\right)\alpha_{j}^{E}+\frac{I_{j}+g_{EE}v_{j}-g_{EI}s_{j}-1}{2}{\left(\alpha_{j}^{E}\right)}^{2}\right] \end{array}$$

and

(A11) $$\begin{array}{l} \frac{\partial \alpha_{j}^{I}}{\partial t}=-i\left[\frac{J_{j}+g_{IE}u_{j}-1}{2}+\left(J_{j}+g_{IE}u_{j}+1\right)\alpha_{j}^{I}+\frac{J_{j}+g_{IE}u_{j}-1}{2}{\left(\alpha_{j}^{I}\right)}^{2}\right] \end{array}$$

Substituting Equations (A8) and (A9) into Equations (A1)–(A3) we obtain

(A12) $$\begin{array}{l} q_{i}=\frac{1}{N}\overset{N}{\underset{j=1}{\sum }}A_{ij}^{IE}\int_{-\infty }^{\infty }h\left(I_{j}\right)H\left(\alpha_{j}^{E}\left(I_{j},t\right);n\right)\;dI_{j} \end{array}$$

(A13) $$\begin{array}{l} r_{i}=\frac{1}{N}\overset{N}{\underset{j=1}{\sum }}A_{ij}^{EE}\int_{-\infty }^{\infty }h\left(I_{j}\right)H\left(\alpha_{j}^{E}\left(I_{j},t\right);n\right)\;dI_{j} \end{array}$$

(A14) $$\begin{array}{l} s_{i}=\frac{1}{N}\overset{N}{\underset{j=1}{\sum }}A_{ij}^{EI}\int_{-\infty }^{\infty }g\left(J_{j}\right)H\left(\alpha_{j}^{I}\left(J_{j},t\right);n\right)\;dJ_{j} \end{array}$$

where *H* is given by Equation (36). Using standard properties of the Lorentzian one can perform the integrals in Equations (A12)–(A14) and defining $z_{j}^{E}\left(t\right)\equiv {\bar{\alpha }}_{j}^{E}\left(I_{0}+i\Delta ,t\right)$ and $z_{j}^{I}\left(t\right)\equiv {\bar{\alpha }}_{j}^{I}\left(J_{0}+i\Delta ,t\right)$ we have

(A15) $$\begin{array}{l} q_{i}=\frac{1}{N}\overset{N}{\underset{j=1}{\sum }}A_{ij}^{IE}H\left(z_{j}^{E}\left(t\right);n\right) \end{array}$$

(A16) $$\begin{array}{l} r_{i}=\frac{1}{N}\overset{N}{\underset{j=1}{\sum }}A_{ij}^{EE}H\left(z_{j}^{E}\left(t\right);n\right) \end{array}$$

(A17) $$\begin{array}{l} s_{i}=\frac{1}{N}\overset{N}{\underset{j=1}{\sum }}A_{ij}^{EI}H\left(z_{j}^{I}\left(t\right);n\right) \end{array}$$

Evaluating Equation (A10) at *I*_*j*_ = *I*_0_ + *i*Δ and Equation (A11) at *J*_*j*_ = *J*_0_ + *i*Δ we obtain

(A18) $$\begin{array}{l} \frac{dz_{j}^{E}}{dt}=\frac{\left(iI_{0}-\Delta \right){\left(1+z_{j}^{E}\right)}^{2}-i{\left(1-z_{j}^{E}\right)}^{2}}{2}+\frac{i{\left(1+z_{j}^{E}\right)}^{2}\left(g_{EE}v_{j}-g_{EI}s_{j}\right)}{2} \end{array}$$

(A19) $$\begin{array}{l} \frac{dz_{j}^{I}}{dt}=\frac{\left(iJ_{0}-\Delta \right){\left(1+z_{j}^{I}\right)}^{2}-i{\left(1-z_{j}^{I}\right)}^{2}}{2}+\frac{i{\left(1+z_{j}^{I}\right)}^{2}g_{IE}u_{j}}{2} \end{array}$$

for *j* = 1, 2, …*N*. Equations (A15)–(A19) are Equations (42)–(46) in Section 2.4.
